# The distinct category of healthcare associated bloodstream infections

**DOI:** 10.1186/1471-2334-12-85

**Published:** 2012-04-09

**Authors:** Ryan Lenz, Jenine R Leal, Deirdre L Church, Daniel B Gregson, Terry Ross, Kevin B Laupland

**Affiliations:** 1 Department of Medicine, University of Calgary and Alberta Health Services, Calgary, Alberta, Canada; 2 Infection Prevention & Control - Surveillance, Alberta Health Services, Calgary, Alberta, Canada; 3 Department of Pathology and Laboratory Medicine, University of Calgary and Alberta Health Services, Calgary, Alberta, Canada; 4 Division of Microbiology, Calgary Laboratory Services, Calgary, Alberta, Canada; 5 Critical Care Medicine Peter Lougheed Centre, 3500-26th Street NE, Calgary, Alberta T1Y 6J4, Canada

## Abstract

**Background:**

Bloodstream infections (BSI) have been traditionally classified as either community acquired (CA) or hospital acquired (HA) in origin. However, a third category of healthcare-associated (HCA) community onset disease has been increasingly recognized. The objective of this study was to compare and contrast characteristics of HCA-BSI with CA-BSI and HA-BSI.

**Methods:**

All first episodes of BSI occurring among adults admitted to hospitals in a large health region in Canada during 2000-2007 were identified from regional databases. Cases were classified using a series of validated algorithms into one of HA-BSI, HCA-BSI, or CA-BSI and compared on a number of epidemiologic, microbiologic, and outcome characteristics.

**Results:**

A total of 7,712 patients were included; 2,132 (28%) had HA-BSI, 2,492 (32%) HCA-BSI, and 3,088 (40%) had CA-BSI. Patients with CA-BSI were significantly younger and less likely to have co-morbid medical illnesses than patients with HCA-BSI or HA-BSI (p < 0.001). The proportion of cases in males was higher for HA-BSI (60%; p < 0.001 vs. others) as compared to HCA-BSI or CA-BSI (52% and 54%; p = 0.13). The proportion of cases that had a poly-microbial etiology was significantly lower for CA-BSI (5.5%; p < 0.001) compared to both HA and HCA (8.6 vs. 8.3%). The median length of stay following BSI diagnosis 15 days for HA, 9 days for HCA, and 8 days for CA (p < 0.001). Overall the most common species causing bloodstream infection were *Escherichia coli, Staphylococcus aureus*, and *Streptococcus pneumoniae*. The distribution and relative rank of importance of these species varied according to classification of acquisition. Twenty eight day all cause case-fatality rates were 26%, 19%, and 10% for HA-BSI, HCA-BSI, and CA-BSI, respectively (p < 0.001).

**Conclusion:**

Healthcare-associated community onset infections are distinctly different from CA and HA infections based on a number of epidemiologic, microbiologic, and outcome characteristics. This study adds further support for the classification of community onset BSI into separate CA and HCA categories.

## Background

Bloodstream infections (BSI) are an important cause of morbidity and mortality. Traditionally these have been classified into either community acquired (CA) or hospital acquired (HA) infections for epidemiological and infection prevention and control purposes [1]. Nosocomial or hospital acquired infections are commonly defined as those that have onset after admission or are associated with acquisition in the hospital environment, whereas community-acquired infections have been defined as those present or incubating at the time of hospital admission [1]. However, in recent years, there has been a shift in the delivery of healthcare services such that increasingly complex medical services are being provided in the community environment [2]. As a result, community based patients may now present to hospital with infections that share many characteristics with HA infections [3,4].

In 2002, Freidman *et al. *proposed the sub-classification of community onset BSI (obtained as outpatients or identified within 48 hours of hospital admission) into CA and healthcare associated (HCA) BSI [5]. The HCA infections were identified with recent hospital admission or exposure to significant medical care in a community or outpatient setting, while CA infections described other community onset BSI that did not have significant prior healthcare exposure [5]. It was observed that among a study cohort of 504 patients, as compared to CA-BSI, HCA-BSI had significantly more associated co-morbid disease, increased risk for antimicrobial resistance, and higher mortality. Since then, a limited number of other studies have been conducted that further support the value of this new classification category [6-10]. However, several studies conducted to date have been limited by either relatively small sample size and therefore power to detect important differences in categories or have been conducted in selected populations or university based hospitals which may limit broad generalization of results. The objective of this study was to assess the epidemiology and outcome of BSI infections by acquisition classification among all adults admitted to hospitals in a large Canadian population.

## Methods

### Patient population

This study utilized an inception cohort design. The Calgary Zone of Alberta Health Services (formerly known as the Calgary Health Region) is a fully integrated, publicly funded health system that provides virtually all medical and surgical care to the residents of the cities of Calgary and Airdrie and a large surrounding area including a number of smaller towns and communities (population ~ 1.2 million). All Calgary Zone adult (≥ 18 years) residents admitted to one of the three adult major acute care centres in the region (representing > 95% of all acute care admissions) with first episodes of BSI during January 1^st^, 2000 and December 31^st^, 2007 were included in this study. This study was approved by the Conjoint Health Research Ethics Board at the University of Calgary.

### Study protocol

All study subjects were identified using the Electronic Surveillance System (ESS) Database [11]. The ESS Database was developed by a multidisciplinary group of microbiologists, infectious disease specialists, and information technology and quality and safety experts and has registered all residents of the Calgary Zone with BSI since 2000. This database has been developed through linkages between regional microbiology and acute care hospital administrative databases with validated algorithms used to define incident episodes of BSI and allow their classification as either CA, HCA, or HA. Although patients managed in the community are included in the ESS, detailed clinical data is only available for those admitted to hospital. Only patients admitted to hospital were included in this study.

### Definitions

A BSI in the ESS database is defined as the isolation of a pathogen from ≥ 1 set of blood cultures, or ≥ 2 sets of blood cultures drawn within 5 days for organisms commonly associated with contamination including diptheroids, *Bacillus *spp., *Propionibacterium *spp, coagulase negative-staphylococci, viridans group streptococci, and micrococci [12]. Patients with more than one species isolated from blood within 48 hours were considered to have polymicrobial BSI. Hospital acquired BSI was defined as a first positive blood culture ≥ 48 hours after hospital admission or within 48 hours of discharge from hospital. If transferred from another regional acute care institution then the duration of admission was calculated from admission time at the first hospital. A HCA-BSI was defined as first culture obtained < 48 hours of admission and at least one of: 1) visit to a hospital clinic or emergency department within the prior 5-30 days before onset of BSI; 2) hospitalization at a Calgary Zone acute care hospital for 2 or more days within the prior 90 days before BSI; 3) sample submitted for culture from a nursing home or long term care facility; 4) receipt of dialysis; or 5) active cancer. Community-acquired infections are those with first positive culture obtained < 48 hours of admission and not fulfilling criteria for healthcare associated classification. The Charlson score, a weighted index of 17 co-existing medical conditions, was used as the measure of co-morbid illness and was derived from discharge codes using algorithms previously developed and validated in Calgary [13,14].

### Data management and statistical analysis

Analysis was performed using Stata version 11.2 (Stata Corp, College Station, TX). To avoid the assessment of multiple outcomes for a single patient, only the first admission per patient was included in this study. Non-normally distributed variables were reported as medians with inter-quartile ranges (IQR) and compared using the Mann-Whitney U test. Differences in proportions among categorical data were assessed using Fisher's exact test for pair-wise comparisons and the chi squared test for multiple groups. P-values less than 0.05 were considered to be significant.

## Results

### Patients and acquisition classification

During the eight year study period, 7,712 adult residents had first admissions associated with an incident BSI. Of the studied inpatient population, 2,132 (28%) were HA-BSI, 2,492 (32%) HCA-BSI, and 3,088 (40%) were CA-BSI. Among patients with HA-BSI, the median time to positive blood culture after admission was 10.4 (IQR, 4.8-20.2) days. Among the group of patients classified as HCA-BSI (n = 2,492), 1,164 (47%) had been admitted to an acute care hospital for two or more days within the prior 90 days before BSI, 714 (29%) attended a hospital clinic or emergency department within the prior 5-30 days before BSI, 584 (23%) were cancer patients, 395 (16%) were residents of a nursing home or long term care facility, and 170 (7%) were hemodialysis patients. A number of demographics and clinical characteristics were different in each category of acquisition classification as shown in Table 1.

**Table 1:** Characteristics of admitted adults with incident episodes of bloodstream infection according to acquisition classification, Calgary, 2000-2007

	Hospital Acquired (HA-BSI)	Healthcare-Associated (HCA-BSI)	Community-Acquired (CA-BSI)	HA vs HCA *P*	HA vs CA *P*	HCA vs CA *p*
Age (IQR) years	68.0 (53.1-78.0)	70.8 (54.9-81.0)	61.2 (45.7-75.0)	< 0.001	< 0.001	< 0.001

Male (%)	1,282 (60%)	1,284 (52%)	1,654 (54%)	< 0.001	< 0.001	0.131

Poly-microbial	183 (9%)	206 (8%)	170 (6%)	0.710	< 0.001	< 0.001

Median Charlson (IQR) score	2 (1-4)	2 (0-4)	1 (0-2)	< 0.001	< 0.001	< 0.001

Charlson category				< 0.001	< 0.001	< 0.001

0	422 (20%)	633 (25%)	1,525 (49%)			

1	327 (15%)	433 (17%)	687 (22%)			

2	453 (21%)	535 (21%)	355 (12%)			

≥ 3	928 (44%)	890 (36%)	520 (17%)			

In-hospital all-cause crude mortality	639/2,132 (30%)	449/2,492 (18%)	335/3,088 (11%)	< 0.001	< 0.001	< 0.001

### Microbiology

Overall the most common species causing bloodstream infection was *Escherichia coli, Staphylococcus aureus*, and *Streptococcus pneumoniae*. The distribution and relative rank of prevalence of these species varied according to classification of acquisition as shown in Table 2. Among *Escherichia coli *isolates, rates of ciprofloxacin resistance were significantly (p < 0.001) higher among HA-BSI (52/318; 16%) and HCA-BSI (104/649; 16%) as compared to CA-BSI (83/959; 9%) and this was also observed for resistance to ceftriaxone (HA 22/316; 7% vs. HCA 34/646; 5% vs. CA 22/953; 2%; p < 0.001). The proportion of methicillin-resistant *Staphylococcus aureus *(MRSA) isolates was similar between HA-BSI and HCA-BSI (97/607; 16% vs. 75/475; 16%; p = 1.0), while this was significantly lower in CA-BSI (27/366; 7%; p < 0.001). The overall rate of penicillin resistance in *S. pneumoniae *was low (41/658; 6%) and was not different among the acquisition categories (p = 0.83).

**Table 2:** Ten most common isolates causing bloodstream infection by acquisition classification, Calgary, 2000-2007

Species	Overall (%)	HA (%)	HCA (%)	CA (%)
*Escherichia coli*	2,052 (24)	340 (14)	689 (25)	1,023 (31)

*Staphylococcus aureus*	1,448 (17)	607 (26)	475 (17)	366 (11)

*Streptococcus pneumoniae*	686 (8)	39 (2)	177 (6)	470 (14)

Coagulase negative staphylococcus	497 (6)	241 (10)	160 (6)	96 (3)

*Klebsiella pneumoniae*	482 (6)	135 (6)	184 (7)	163 (5)

*Enterococcus faecalis*	286 (3)	115 (5)	103 (4)	68 (2)

*Bacteroides fragilis*	262 (3)	86 (4)	79 (3)	97 (3)

Group A streptococcus	244 (3)	18 (4)	75 (3)	151 (5)

*Pseudomonas aeruginosa*	220 (3)	105 (4)	83 (3)	32 (1)

Group B streptococcus	168 (2)	27 (1)	53 (2)	88 (3)

Other	2,039 (24)	637 (27)	671 (24)	731 (22)

### Outcomes

The overall median length of hospital stay was 11 days (IQR, 6 to 27 days). There was a significant (p < 0.001) difference in the median length of stay following BSI diagnosis among HA-BSI patients (15 days, IQR, 6 to 36) as compared to HCA (9 days, IQR, 5 to 18) and CA-BSI (8 days, IQR 4 to 14). The overall all-cause inpatient case-fatality rate was 1,423/7,712 (18%). Significantly more in-hospital deaths were seen in patients with HA-BSI (639/2,132; 30%) than in patients with HCA-BSI (449/2,492; 18%; p < 0.001) which in turn had a higher mortality rate than CA-BSI (335/3,088; 11%; p < 0.001). Similarly, 28-day all cause mortality was highest in HA-BSI (554/2,132; 26%), compared to HCA-BSI (460/2,492; 18%) and CA-BSI (307/3,088; 10%). Differences were significant (p < 0.001) between each category.

## Discussion

In this study we found that significant differences between the three acquisition classifications in relation to demographics, co-morbidities, microbiology, and hospital course and mortality outcomes. On one hand, the characteristics of HCA-BSI were similar to HA-BSI as regards age, the proportion of MRSA, and poly-microbial infections. On the other hand, HCA-BSI resembled CA-BSI in length of stay and the proportion of males. However, features of HCA-BSI were intermediate between HA and CA-BSI as regards infecting species, distribution of co-morbidities, and mortality (Tables 1 and 2). Overall, we consider HCA-BSI to represent a category that is intermediate in clinical, microbiological, and outcome characteristics between HA-BSI and CA-BSI among patients requiring admission to hospital. This study confirms HCA-BSI as a distinct acquisition category and further supports its use in surveillance and in epidemiology studies of infection.

We modeled our study definitions based on those proposed by Freidman *et al *[5] and found generally similar findings with their landmark study. Among their cohort of 504 patients with BSI, they found that *E. coli *and *S. pneumoniae *were most common in CA, and that *S. aureus *was the most common in HA and HCA disease. They reported that MRSA occurred with similar frequency among HCA and HA but much lower in CA-BSI. Our results are consistent with these findings with the notable exception that *E. coli *was found to be the most common cause of HCA-BSI in our study (Table 2). In addition, they found that the distribution of co-morbid illnesses was similar among HA- and HCA-BSI and higher than in CA-BSI, and that hospital length of stay was much longer in HA-BSI but similar in HCA- and CA-BSI. Furthermore, they observed that in-hospital mortality rates were higher in HA-BSI (30%), as compared to HCA-BSI (20%), and CA-BSI (13%). Although clinically significant, they did not find statistically significant difference between HCA-BSI and CA-BSI in-hospital mortality that was likely related to limited statistical power. While our study mirrors these differences in mortality rates between the acquisition categories, our more than 10-fold larger sample size had adequate power to detect statistically significant differences in mortality outcome between each acquisition category both with respect to in-hospital death as well as 28-day mortality.

A number of other studies have investigated alternate definitions for community onset BSI or have evaluated HCA-BSI in different populations of patients [3]. Siegman-Igra proposed four main different categories (with five other subgroups) for community onset BSI and found a number of different demographic, clinical, and outcome differences among 569 patients admitted to a large university hospital in Tel Aviv in 1997 [3].

Son *et al *investigated more than 1,144 BSI isolates obtained from 9 university hospitals in Korea [6]. As in our study, they found that *E. coli *was the most common pathogen in HCA- and CA-BSI and *S. aureus *in HA-BSI, that co-morbidities were most prevalent in HA- and HCA-BSI, and that there was a decreasing risk for 30-day mortality from HA- (23%), to HCA- (18%) to CA-BSI (10%). Valles *et al *investigated 1,157 episodes of BSI among adults admitted to three Spanish teaching hospitals and found that as compared to patients with CA-BSI, patients with HCA-BSI were more likely to have MRSA infection and suffered significantly higher mortality [7]. McDonald and colleagues investigated 466 BSI at three North Carolina hospitals and found that as compared to CA-BSI, HCA-BSI was an independent predictor of inadequacy of treatment and this was most strongly related to prior hospitalization within the prior 90 days [8]. Shorr *et al *investigated a cohort of 6,697 patients admitted to 59 hospitals in the United States and found that HCA-BSI had more co-morbid illness and a higher proportion of MRSA as compared to CA-BSI [9]. In addition, they found that the length of stay was similar between HCA-BSI and CA-BSI but less than for HA-BSI, and that the mortality rates for CA-BSI of 10% was significantly less than for HCA-BSI (15%) and HA-BSI (15%). It is notable that they did not find a difference in mortality among HA-BSI and HCA-BSI and this may be explained in part by their focus on inclusion of patients with cultures obtained in the first five days after admission to hospital. Kollef and colleagues compared HCA-BSI and CA-BSI among 1,143 patients admitted to 7 American hospitals and found that patients with HCA-BSI were older, were more likely to be male, presented with higher severity of illness, had longer lengths of stay, and were more likely to die [10]. They did not examine HA-BSI. A number of other studies have investigated the category of HCA-BSI but these have been limited to restricted populations or infecting etiologies [15-18].

Despite the growing body of evidence supporting the unique nature of HCA-BSI, there is still failure to universally recognize this classification in contemporary BSI definitions and literature, including the current Centers for Disease Control definition for healthcare associated (previously nosocomial) infections [19]. Using these definitions, BSI detected after 48 hours of hospitalization is designated HA, with the remainder of all episodes classified CA-BSI by default, including those that would fall into the proposed HCA-BSI category. An exception is the MRSA classification, which includes a distinct healthcare associated - community onset category analogous to HCA-BSI [20].

Identification of the HCA-BSI category has a number of implications for surveillance. Hospital-acquired infections are an important measure of quality of care. With the shift towards increasing delivery of complex care in the community setting, decreasing numbers of cases of HA-BSI may be falsely viewed as a marker of success if HCA-BSI's are not tracked to ensure that there is not a concomitant increase in these infections. Similarly, identification of emerging resistant organisms in the community may be falsely attributed to some community based factor (such as travel, food exposures, environmental contamination) if a healthcare associated linkage is not recognized [21]. Perhaps most importantly, failure to recognize that HCA-BSI have higher rates of resistance than CA-BSI may lead to adverse outcomes as a result of increased risk for treatment failure [6,8].

While our study benefits from large sample size and comprehensive inclusion of all cases occurring in a large well-defined population there are some limitations that are noteworthy. We obtained all of our patient information from our Electronic Surveillance System that is based solely on source data available from regional and provincial databases [11]. We did not have data on specialized home care, immuno-suppressive medication use, and ambulatory parenteral antibiotherapy. As a result, we had to make minor modifications of the definitions of Friedman et al [5] and did not perform a case-by-case individual chart or patient review in this study. Thus, it is possible that our assignment of patients to acquisition categories may not have been as precise as if we had performed a case-by-case individual review and subsequently patients may have been misclassified. However, our classification of acquisition by the ESS has been previously demonstrated to be highly accurate based on a retrospective comparative review of 306 patients in development [11], and a further 300 plus cases in validation (manuscript in preparation). It is important to note that while our results are comparable to the study by Friedman because of the similar definitions used [5], generalization and comparison with other studies that used different definitions should be done cautiously [3,6-10]. Another limitation of our study is that we did not have information on treatments prescribed as it would be interesting to evaluate whether the HCA-BSI category were at increased risk for inadequate empiric antibiotic therapy. While we report selected resistance rates in this study, we do not present overall group specific resistance rates and therefore may not be recognizing further important differences in acquisition categories. A third limitation is that we did not assess sources of infection, and their may be significant differences in the distribution of foci of infections among the different acquisition classifications. Finally, like all of the other studies conducted to date, we only included patients admitted to hospital. The possibility does exist that HCA-BSI and CA-BSI differ further in this non-admitted population.

## Conclusions

In this study we found that HCA-BSI were a distinct category of BSI that have features intermediate between CA-BSI and HCA-BSI. These data add further support for the classification of community onset BSI into CA-BSI and HCA-BSI in infectious diseases surveillance and inclusion in guidelines guiding empiric antimicrobial therapies in patients presenting to hospital with infectious diseases.

## Competing interests

The authors declare that they have no competing interests.

## Authors' contributions

RL contributed to study design and drafted the manuscript. JRL, DBG, DLC, and TR contributed to study design and data collection. KBL conceived the study and contributed to study design, data collection, analysis, and drafting of the manuscript. All authors contributed to critical revision and approval of the final manuscript.

## Pre-publication history

The pre-publication history for this paper can be accessed here:

http://www.biomedcentral.com/1471-2334/12/85/prepub

## References

[B1] Garner JS Jarvis WR Emori TG Horan TC Hughes JM CDC definitions for nosocomial infections, 1988 Am J Infect Control 1988 16 3 128 140 10.1016/0196-6553(88)90053-3 2841893

[B2] Jarvis WR Infection control and changing health-care delivery systems Emerg Infect Dis 2001 7 2 170 173 10.3201/eid0702.010202 11294699 PMC2631740

[B3] Siegman-Igra Y Fourer B Orni-Wasserlauf R Golan Y Noy A Schwartz D Giladi M Reappraisal of community-acquired bacteremia: a proposal of a new classification for the spectrum of acquisition of bacteremia Clin Infect Dis 2002 34 11 1431 1439 10.1086/339809 12015688

[B4] Diekema DJ Beekmann SE Chapin KC Morel KA Munson E Doern GV Epidemiology and outcome of nosocomial and community-onset bloodstream infection J Clin Microbiol 2003 41 8 3655 3660 10.1128/JCM.41.8.3655-3660.2003 12904371 PMC179863

[B5] Friedman ND Kaye KS Stout JE McGarry SA Trivette SL Briggs JP Lamm W Clark C MacFarquhar J Walton AL Health care-associated bloodstream infections in adults: a reason to change the accepted definition of community-acquired infections Ann Intern Med 2002 137 10 791 797 12435215 10.7326/0003-4819-137-10-200211190-00007

[B6] Son JS Song JH Ko KS Yeom JS Ki HK Kim SW Chang HH Ryu SY Kim YS Jung SI Bloodstream infections and clinical significance of healthcare-associated bacteremia: a multicenter surveillance study in Korean hospitals J Korean Med Sci 2010 25 7 992 998 10.3346/jkms.2010.25.7.992 20592888 PMC2890898

[B7] Valles J Calbo E Anoro E Fontanals D Xercavins M Espejo E Serrate G Freixas N Morera MA Font B Bloodstream infections in adults: importance of healthcare-associated infections J Infect 2008 56 1 27 34 10.1016/j.jinf.2007.10.001 18022242

[B8] McDonald JR Friedman ND Stout JE Sexton DJ Kaye KS Risk factors for ineffective therapy in patients with bloodstream infection Arch Intern Med 2005 165 3 308 313 10.1001/archinte.165.3.308 15710794

[B9] Shorr AF Tabak YP Killian AD Gupta V Liu LZ Kollef MH Healthcare-associated bloodstream infection: A distinct entity? Insights from a large U.S. database Crit Care Med 2006 34 10 2588 2595 10.1097/01.CCM.0000239121.09533.09 16915117

[B10] Kollef MH Zilberberg MD Shorr AF Vo L Schein J Micek ST Kim M Epidemiology, microbiology and outcomes of healthcare-associated and community-acquired bacteremia: a multicenter cohort study J Infect 2011 62 2 130 135 10.1016/j.jinf.2010.12.009 21195110

[B11] Leal J Gregson DB Ross T Flemons WW Church DL Laupland KB Development of a novel electronic surveillance system for monitoring of bloodstream infections Infect Control Hosp Epidemiol 2010 31 7 740 747 10.1086/653207 20470039

[B12] Yokoe DS Anderson J Chambers R Connor M Finberg R Hopkins C Lichtenberg D Marino S McLaughlin D O'Rourke E Simplified surveillance for nosocomial bloodstream infections Infect Control Hosp Epidemiol 1998 19 9 657 660 10.1086/647894 9778164

[B13] Quan H Parsons GA Ghali WA Validity of information on comorbidity derived rom ICD-9-CCM administrative data Med Care 2002 40 8 675 685 10.1097/00005650-200208000-00007 12187181

[B14] Charlson ME Pompei P Ales KL MacKenzie CR A new method of classifying prognostic comorbidity in longitudinal studies: development and validation J Chronic Dis 1987 40 5 373 383 10.1016/0021-9681(87)90171-8 3558716

[B15] Lesens O Hansmann Y Brannigan E Hopkins S Meyer P O'Connel B Prevost G Bergin C Christmann D Healthcare-associated Staphylococcus aureus bacteremia and the risk for methicillin resistance: is the Centers for Disease Control and Prevention definition for community-acquired bacteremia still appropriate? Infect Control Hosp Epidemiol 2005 26 2 204 209 10.1086/502527 15756893

[B16] Liao CH Chen SY Chang SC Hsueh PR Hung CC Chen YC Characteristics of community-acquired and health care-associated Staphylococcus aureus bacteremia in patients treated at the emergency department of a teaching hospital Diagn Microbiol Infect Dis 2005 53 2 85 92 10.1016/j.diagmicrobio.2005.06.009 16168612

[B17] Cheong HS Kang CI Kwon KT Heo ST Wi YM Kim ES Lee JS Ko KS Chung DR Lee NY Clinical significance of healthcare-associated infections in community-onset Escherichia coli bacteraemia J Antimicrob Chemother 2007 60 6 1355 1360 10.1093/jac/dkm378 17923453

[B18] Ortega M Almela M Soriano A Marco F Martinez JA Munoz A Penarroja G Mensa J Bloodstream infections among human immunodeficiency virus-infected adult patients: epidemiology and risk factors for mortality Eur J Clin Microbiol Infect Dis 2008 27 10 969 976 10.1007/s10096-008-0531-5 18449581

[B19] Horan TC Andrus M Dudeck MA CDC/NHSN surveillance definition of health care-associated infection and criteria for specific types of infections in the acute care setting Am J Infect Control 2008 36 5 309 332 10.1016/j.ajic.2008.03.002 18538699

[B20] Klevens RM Morrison MA Nadle J Petit S Gershman K Ray S Harrison LH Lynfield R Dumyati G Townes JM Invasive methicillin-resistant Staphylococcus aureus infections in the United States Jama 2007 298 15 1763 1771 10.1001/jama.298.15.1763 17940231

[B21] Laupland KB Church DL Vidakovich J Mucenski M Pitout JD Community-onset extended-spectrum beta-lactamase (ESBL) producing Escherichia coli: Importance of international travel J Infect 2008 10.1016/j.jinf.2008.09.034 18990451

